# Describing Art – An Interdisciplinary Approach to the Effects of Speaking on Gaze Movements during the Beholding of Paintings

**DOI:** 10.1371/journal.pone.0102439

**Published:** 2014-12-10

**Authors:** Christoph Klein, Juliane Betz, Martin Hirschbuehl, Caroline Fuchs, Barbara Schmiedtová, Martina Engelbrecht, Julia Mueller-Paul, Raphael Rosenberg

**Affiliations:** 1 School of Psychology, Bangor University, Bangor, United Kingdom; 2 Department for Child and Adolescent Psychiatry, Psychotherapy and Psychosomatics, University of Freiburg, Freiburg, Germany; 3 Institute for European Art History, University of Heidelberg, Heidelberg, Germany; 4 Department of Art History and Cognitive Research Platform, University of Vienna, Vienna, Austria; 5 Institute for German as a Foreign Language Philology, University of Heidelberg, Heidelberg, Germany; University of Leicester, United Kingdom

## Abstract

Ever since the Renaissance speaking about paintings has been a fundamental approach for beholders, especially experts. However, it is unclear whether and how speaking about art modifies the way we look at it and this was not yet empirically tested. The present study investigated to the best of our knowledge for the first time in what way speaking modifies the patterns of fixations and gaze movements while looking at paintings. Ninety nine university students looked at four paintings selected to cover different art historical typologies for periods of 15 minutes each while gaze movement data were recorded. After 10 minutes, the participants of the experimental group were asked open questions about the painting. Speaking dramatically reduced the duration of fixations and painting area covered by fixations while at the same time increasing the frequencies of fixations, gaze length and the amount of repeated transitions between fixation clusters. These results suggest that the production of texts as well-organised sequences of information, structures the gazes of art beholders by making them quicker, more focused and better connected.

## Introduction

The most radical innovation about visual arts in the Renaissance is, next to the introduction of perspective and the recourse to ancient models, arguably the fact that paintings, sculptures and buildings became the object of a theoretical discourse that had not existed before. Leon Battista Alberti (1404–1472) and Giorgio Vasari (1511–1574) are the most famous among the group of pioneers that shaped a language for speaking about visual arts. Private, princely and (later) public art galleries were places where conversations about the works accompanied their beholding [1]. Many early texts about visual arts were written in the form of discourses between different beholders [2]–[4]. Within this new literature domain, the description of single artworks emerged as an essential technique that provides the translation from the visual medium to the realm of language, thus embedding artworks into language-based discourses. Descriptions of art have become the basis of art criticism as it has been developed since the 18^th^ century and of the history of art as an academic discipline that was founded in the 19^th^ century [5]–[7].

Incidentally it should be noticed that especially since the 18^th^ century paintings were often described using hypothetical gaze movements of virtual beholders in order to uncover the compositional structure of those works of art [8]. In speculating about the gaze movements of virtual beholders, art critics and art historians unintentionally and implicitly touched a field that would decades later evolve as a central method in psychology: The recording of gaze movements to investigate cognitive processes [9], [10]. However, not being in possession of eye trackers and not being interested in cognitive processes as such, most of the art historians were not aware that many of the assumptions they made about the structure of eye movements could not be corroborated empirically.

Until the present, and despite the fact that visitors of museums and professional art historians spend much time explaining and discussing works of art, fundamental questions have never been asked: What happens to the way we look at paintings if we speak about them, rather than just looking at them? Does it change our patterns of attention? How does it change our gaze movements and the correlated cognitive processes?

While there is no direct answer to this question, indirect ones come from linguistics and reveal the close relationship between language and cognitive processing during language-based categorization and matching [11]–[14], memorisation or judging similarity [15], recognition [16], [17] as well as orienting visual attention and gaze towards language-specific components of motion events [18]. However, the impact of language on the structuring of gaze movement behaviour becomes apparent only when speaking is required for a given task. This is what Slobin [19] has meant with his “Thinking for Speaking” hypothesis: The preparation of content for verbalisation in the mind of the speaker is constrained by specific linguistic categories available in the speaker's language system. Speech production is therefore linked in task- and/or language-specific ways to the control of gaze movements [20]–[24]. The aforementioned studies are suggestive of close, albeit differentiated relationships between language and speech production on the one side and the control of visual attention and gaze movements on the other. Potential limitations of these studies with respect to the question of how speaking impacts the way we look at paintings, nevertheless, are the facts that comparatively simple stimuli and tasks (e.g., naming of line drawings of objects) have been used and rather short gaze movement recording periods (typically in the range of seconds) have been undertaken.

Paintings, however, are doubtlessly much more complex. The mean time spent viewing major works of art in “high traffic areas” of the Metropolitan Museum of Art (New York) has been measured at 27.2 seconds [25]. Nevertheless, it is the experience of professional art historians that the viewing time of experts such as artists, art historians or students of art history, especially in groups when speaking with each other can often last for several minutes during which an initial phase of silent contemplation is followed by discourses about the art work under consideration. Such reception of art involves complex interactions between various kinds of long-term memory processes. Some of these processes may use verbal codes (such as the appreciation of familiarity [of style or genre]) others do use them (such as explicit classification [26]). Speaking as the actualisation of language should therefore interact with the reception of art as evidenced through gaze movements.

The process of describing art has not yet been empirically tested but we do know that certain ‘instructions’ influence the way people look at paintings and their gaze movements. Yarbus [27] was the first to test various task instructions (such as estimation of material circumstances of the represented families or their members' ages). He found that his participants fixated different pictorial elements depending on the instruction (e.g., furniture versus faces). Molnar [28], [29] found that participant groups who knew that they were expected to verbally report later on either the semantic content of a painting or its aesthetic quality, fixated the pictorial elements of a painting in significantly different ways (longer fixations in the aesthetic group).

Based on these considerations, the present study aimed at investigating the potential effects of speaking on the beholding of visual art. In this regard, it is important to note that speakers produce texts. Texts are not loose assemblies of words or sentences, but they are organized on the basis of a global structure which allows for coherent progression of information. Because of this attention-focussing and -structuring effects, we predicted that speaking would reduce the paintings' areas covered by fixations, but increase the density of repeated transitions between areas of fixations. Furthermore, we expected this general effect to be more pronounced for representational art as compared to abstract art as the former facilitates the retrieval of verbal codes.

## Methods

### Ethics statement

The present study was conducted in accordance with the Declaration of Helsinki (revised 1983) and local guidelines of the Faculty of Psychology, University of Heidelberg. According to the German University Law at that time (and until 2012), only medical faculties were required to appoint ethics committees for clinical tests, application of medical methods, and applied medical research etc. Therefore, ethical approval was neither required nor obtainable for the present study. Written informed consent was given by all participants who could withdraw at any time during the experiment without further consequences.

### Participants

Subjects were recruited with advertisements that were placed in different institutes of the University of Heidelberg. They were selected for the study on the basis of a telephone interview and an ad hoc art experience questionnaire comprising of eight questions related to formal criteria of art training. Participants with relatively high and relatively low experience with art were randomly assigned to the experimental (with interviews) and control groups (without interviews). Most of the “experienced” subjects were students of the history of art, the other participants were recruited from various other departments within the humanities of the University of Heidelberg to be similar in their capabilities of language expression. From these two groups, data of N = 47 control participants (age: 23.5±2.8; mean art experience score: 8.7) and N = 52 experimental participants (age: 23.9±2.5; mean art experience score: 8.9) were available for statistical analysis.

### Hardware

**Figure 1:**
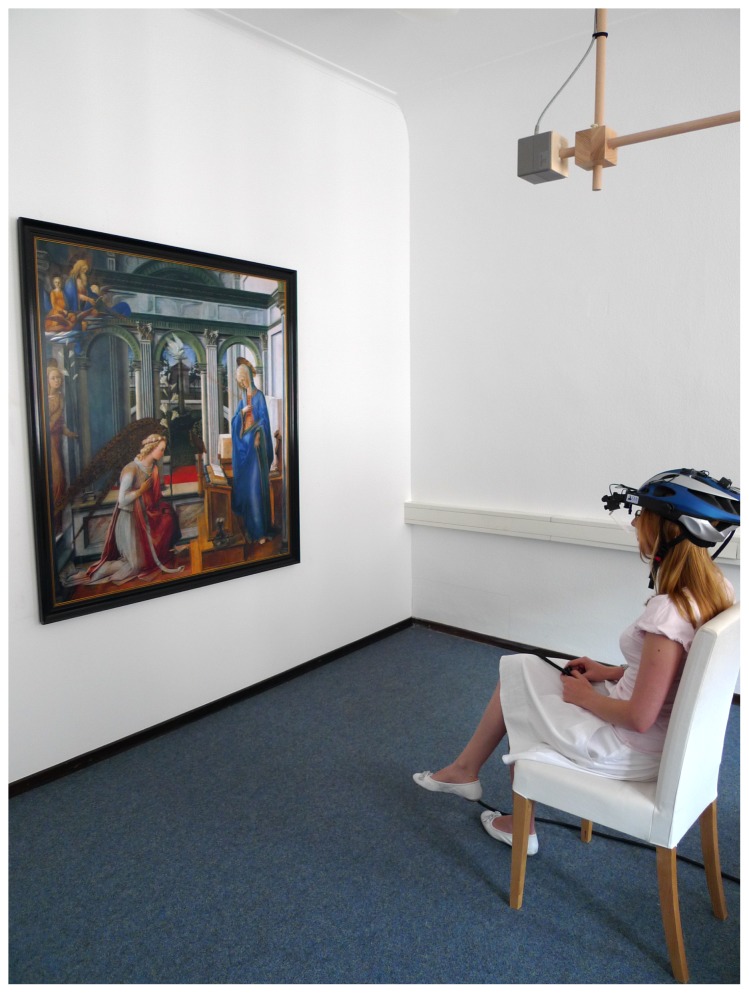
Looking at paintings under fairly “naturalistic” conditions. This figure illustrates the testing conditions in the Heidelberg gaze movement lab. Participants were allowed sit on a chair or walked around within a circle with a radius of 1.2 meter to approximate viewing conditions in art galleries.

Reproductions of highest possible quality in the size of the originals were produced by using large size museum slides (ektachromes), digitally printed on photographic paper (with a Cymbolic Science Lightjet 5000), laminated on a board and set in a wooden frame that was suitable for the specific epoch of the painting. The paintings presented in this study (see **Figs. 1** **–** **4**) were selected according to the following criteria. (1) Filippo Lippi's “Annunciation” (c.1450, Alte Pinakothek, Munich; 135.3 cm * 123.7 cm, thus smaller as the original measuring 203 cm * 186 cm) was chosen as example of representational religious art that requires Christian iconographical background knowledge to understand its meaning and compositional structure. (2) Peter Bruegel's “The Blind Leading the Blind” (1568, Museo Nazionale di Capodimonte, Naples; 84.5 cm * 149.0 cm, thus slightly smaller as the original measuring 86 cm * 154 cm) was selected as a classic example of representational art that has an easy to grasp compositional structure (diagonal fall from left to right) and does not require much background knowledge to understand its meaning (blinds are lead by a blind person and are all to fall). (3) Franz Marc's “Fighting Forms” (1914, Pinakothek der Moderne, Munich; 91.1 cm * 130.6 cm) was chosen as an example of abstract painting that shows a left-to-right orientation (the red form on the left is “attacking” the dark blue form on the right) and might require experience in looking at abstract art for its understanding. The paintings by Bruegel, Lippi, and Marc have a clear left-to-right orientation in common. (4) Vincent van Gogh's “Young Male Peasant” (1889, Peggy Guggenheim Collection, Venice; 49.5 cm * 60.5 cm) was chosen as another piece of representational art, which does not require art expertise knowledge for its understanding and which does not exhibit a left-to-right orientation. This selection of paintings thus varies stimuli according to core determinants of art history (i.e., representational versus abstract; left-right-orientation (present, absent); and specific iconographic knowledge required (yes, no). This selection, however, is not meant to constitute an experimental factorial design, but rather to cover a fundamental diversity of “typologies” that is meaningful in art historical terms. All paintings were presented hanging on one of the walls of the gaze movement laboratory which was approximately 5×5 meters in size. They were mounted immediately before a corresponding testing block and invisible to participants before and after that block.

**Figure 2:**
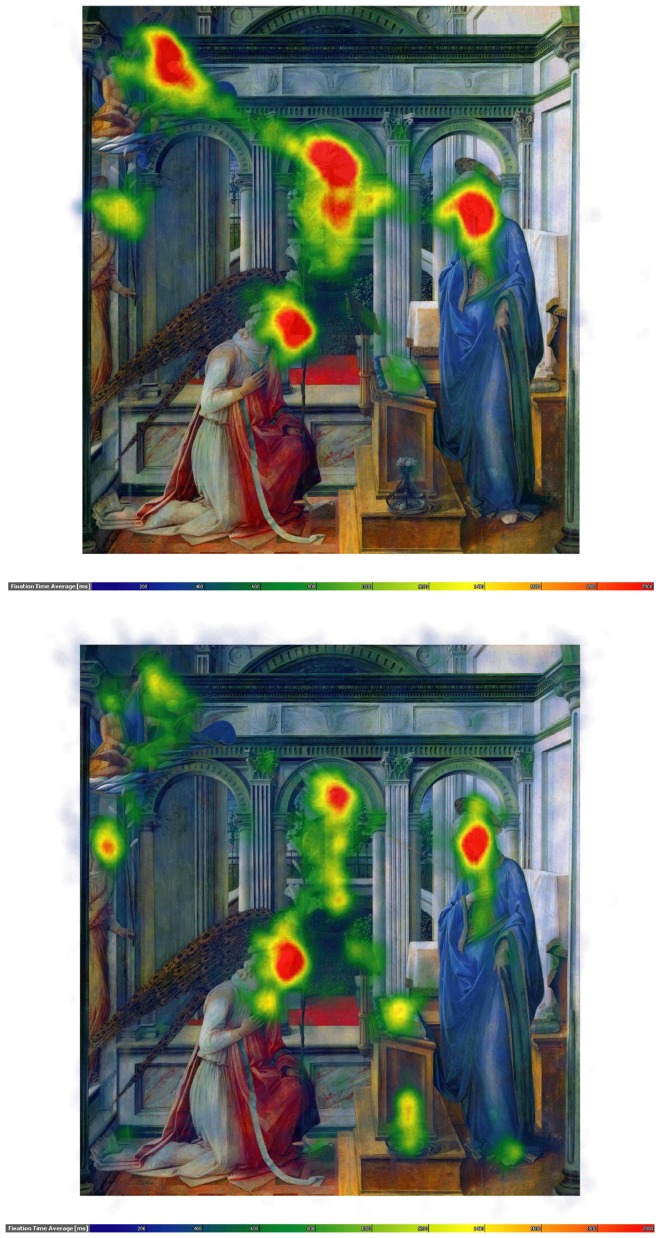
Speaking effects on fixation dispersion and duration – Lippi. Upper part: Heat map showing the total time certain areas of the painting have been fixated by the experimental group during minute 11 to 15. Areas fixated for more than 2,000 ms/minute are displayed in red. Colour scaling is in 200 ms steps. Lower part: Heat map showing the total time certain areas of the painting have been fixated by the control group during minute 11 to 15. Areas fixated for more than 2,000 ms/minute are displayed in red. Colour scaling is in 200 ms steps.

**Figure 3:**
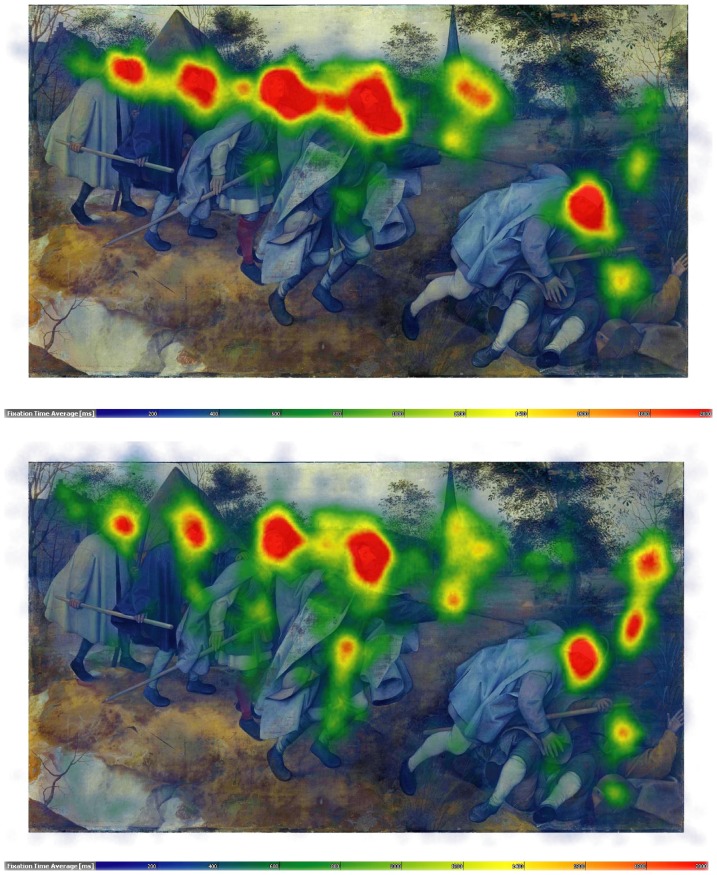
Speaking effects on fixation dispersion and duration – Bruegel. Upper part: Heat map visualising the total time certain areas of the painting have been fixated by the experimental group during minute 11 to 15. Areas fixated for more than 2,000 ms/minute are displayed in red. Colour scaling is in 200 ms steps. Lower part: Heat map visualising the total time certain areas of the painting have been fixated by the control group during minute 11 to 15. Areas fixated for more than 2,000 ms/minute are displayed in red. Colour scaling is in 200 ms steps.

**Figure 4:**
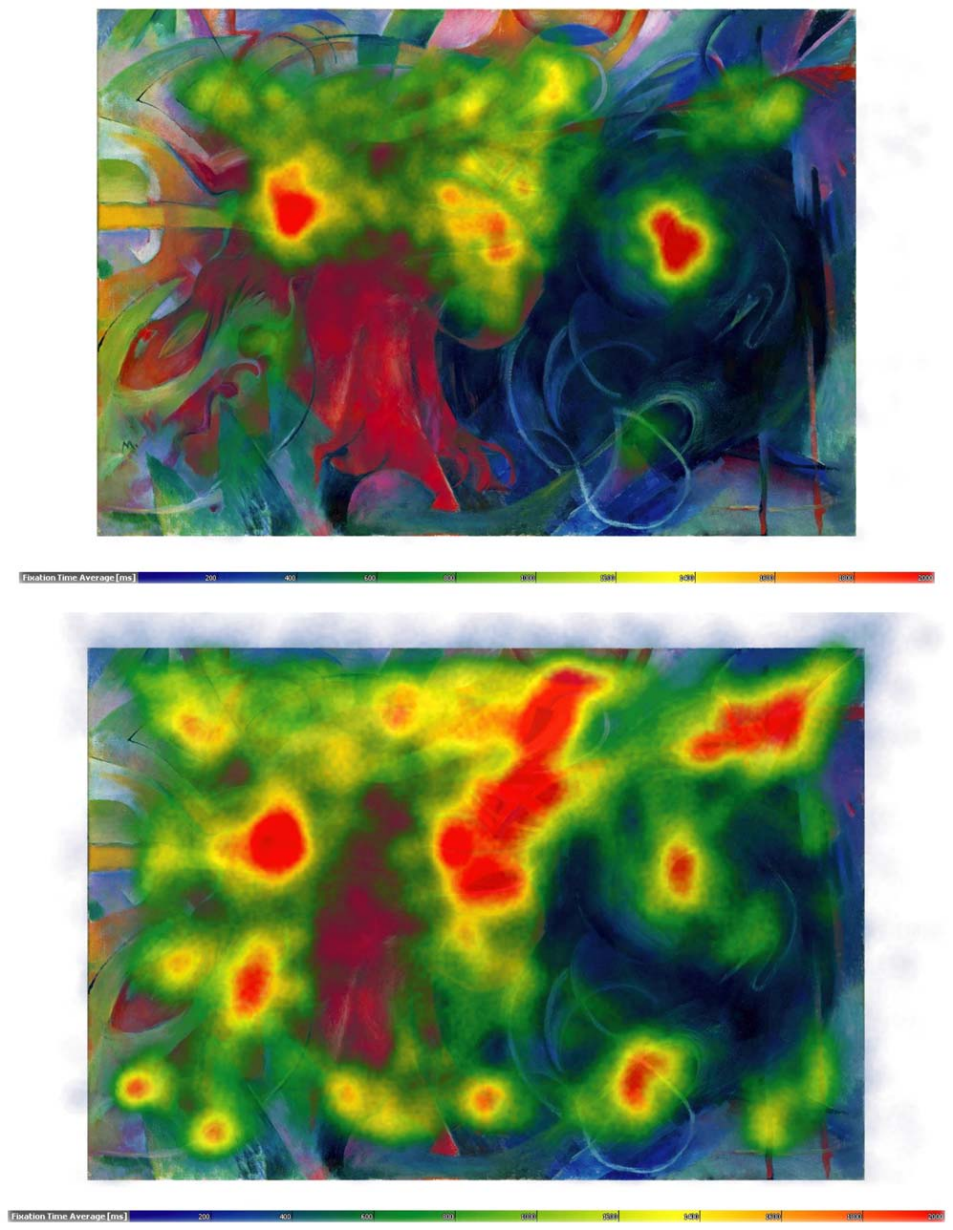
Speaking effects on fixation dispersion and duration – Marc. Heat map showing the total time certain areas of the painting have been fixated by the experimental group during minute 11 to 15. Areas fixated for more than 2,000 ms/minute are displayed in red. Colour scaling is in 200 ms steps. Heat map visualising the total time certain areas of the painting have been fixated by the control group during minute 11 to 15. Areas fixated for more than 2,000 ms/minute are displayed in red. Colour scaling is in 200 ms steps.

Participants sat about two meters in front of the painting (see **Fig. 1**), but were allowed to stand up or even walk up to the painting (the movement of the head was possible within a total radius of 1.2 meter around a Polhemus digitizer with a transmitter box hanging from the ceiling 2.2 meter above the floor and allowing to calculate the precise position of the eye in space). The experimenter sat another two meters behind the participant. Gaze movements were calibrated before each painting and were recorded with the head-mounted iViewX HED-HT system (by SMI, Teltow, Germany), which consists of recording devices and a headset-mounted infrared camera to record right eye movements with a 50 Hz sampling rate using the 'dark pupil' setting. According to our own tests the accuracy of the eye tracker in our setting was at least 0.7 degrees.

### Procedures

All participants were tested individually after having given written informed consent. Visual acuity and colour viewing were determined before the beginning of the experiment, and a 13-dot fixation pattern used to calibrate the iViewX system. The calibration was performed twice: At the beginning and once again after a pause of ca. 10 minutes between the presentation of the second and third painting. The accuracy of the eye tracker in our setting was at least 0.7 degrees. The four paintings were presented in randomised orders. We implemented a free-viewing condition, and participants were instructed to contemplate the paintings for several minutes. While the control group contemplated each painting for 15 min, the experimental group was interviewed right after a 10 min period of silent beholding. The interview consisted of open questions in the following order: "Please describe what you see on the painting!", "How would you interpret the painting?", "Did the painting remind you of something?", and "Did you like the painting?". The aim of the questions was to induce the participants to speak about the paintings for as close as possible to 5 minutes. Depending on their amount of text production the last questions were dropped or the speaking time was partly shorter than 5 minutes. Gaze movement recordings continued whilst participants answered to the interview questions in this particular order. During the interview, the experimenter remained seated behind the participant to avoid gaze contact between participant and experimenter.

### Data analysis

The entire recording period per painting was subdivided into 3 segments of 5 min duration each (with the 3^rd^ segment being up to 2 min shorter in some of the more tight-lipped participants of the experimental group). The data analysis using the self-programmed “EyeTrace” software has been set to define fixations as groups of raw data points within circles of 15 mm diameter (corresponding to 0.86 degrees visual angle at a viewing distance of 1 m) and for minimum durations of 120 ms and outputs the number of fixations per minute in a given data segment, the average duration of fixations in a given data segment and the proportion of a painting's area that is covered by fixations as parameters. Occasional gaze movements outside the painting were thus not analysed. “Heat maps” colour-coding the average sums of fixation durations per painting (from 200 ms (dark blue) to 2,000 ms or more (red) per minute) are shown in **Figs. 2** **–** **5** to illustrate fixation results. Similarly, the gaze movements that link fixations are quantified according to their average length and their relative frequency. Using a “bottom up” approach, we also grouped fixations in fixation “clusters” of specified size defining a cluster of a given radius (depending on the size of the painting and its elements: 20 mm for van Gogh; 60 mm for Bruegel and Lippi; and 120 mm for Marc due to the large-area forms displayed in this painting) as a circle where a certain minimum amount of fixations (at least 1 per minute for van Gogh; 1.3 pm for Bruegel and Lippi; 4 pm for Marc, depending on the sizes of the painting and of the clusters) are to be found, and computed the absolute frequency of cluster transitions in segment that were repeated for at least 0.4 times per minute, the relative frequency of such repeated cluster transitions (relative to the total amount of gaze movements) in segment and the average length of such repeated cluster transitions in segment. Maps showing cluster locations as circles and coding the relative frequencies of cluster transitions by the strengths of the connecting line are shown in **Figs. 6** **–** **9**. Due to the ambiguities associated with the “top down” definition of “regions of interest” (ROI) and the comparison of ROI data across paintings that differ in their gross geometrical structure, we refrained from analysing ROI data. Univariate analyses of variance (ANOVA) were run that distinguished the different PAINTINGS (Lippi, Bruegel, Marc, van Gogh) experimental GROUPS (speaking, no speaking), and the testing SEGMENT (1^st^–5^th^ min, 6^th^–10^th^ min, 11^th^ min-end).

**Figure 5:**
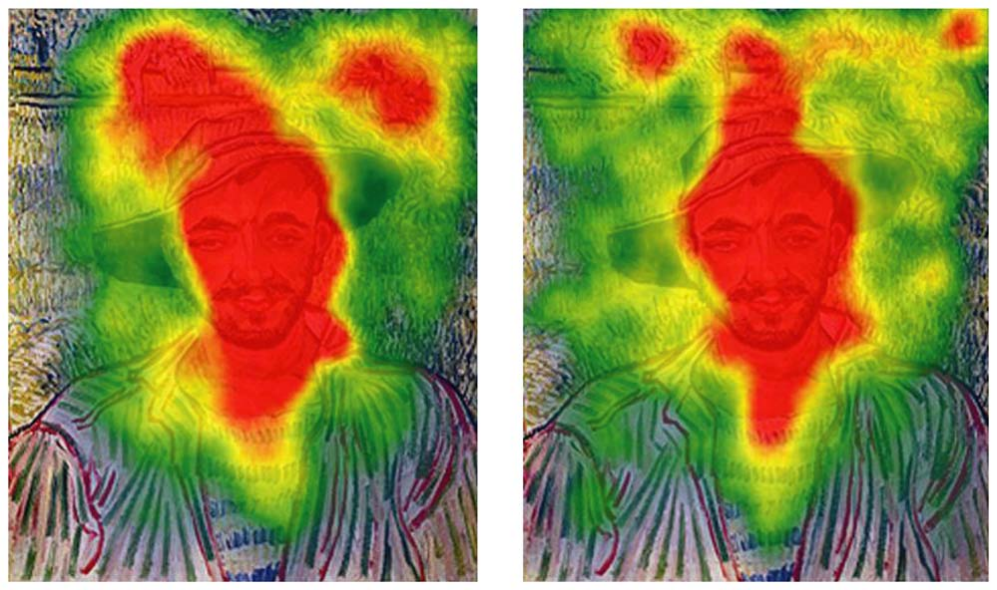
Speaking effects on fixation dispersion and duration – van Gogh. Left side: Heat map visualising the total time certain areas of the painting have been fixated by the experimental group during minute 11 to 15. Areas fixated for more than 2,000 ms/minute are displayed in red. Colour scaling is in 200 ms steps. Right side: Heat map visualising the total time certain areas of the painting have been fixated by the control group during minute 11 to 15. Areas fixated for more than 2,000 ms/minute are displayed in red. Colour scaling is in 200 ms steps.

**Figure 6:**
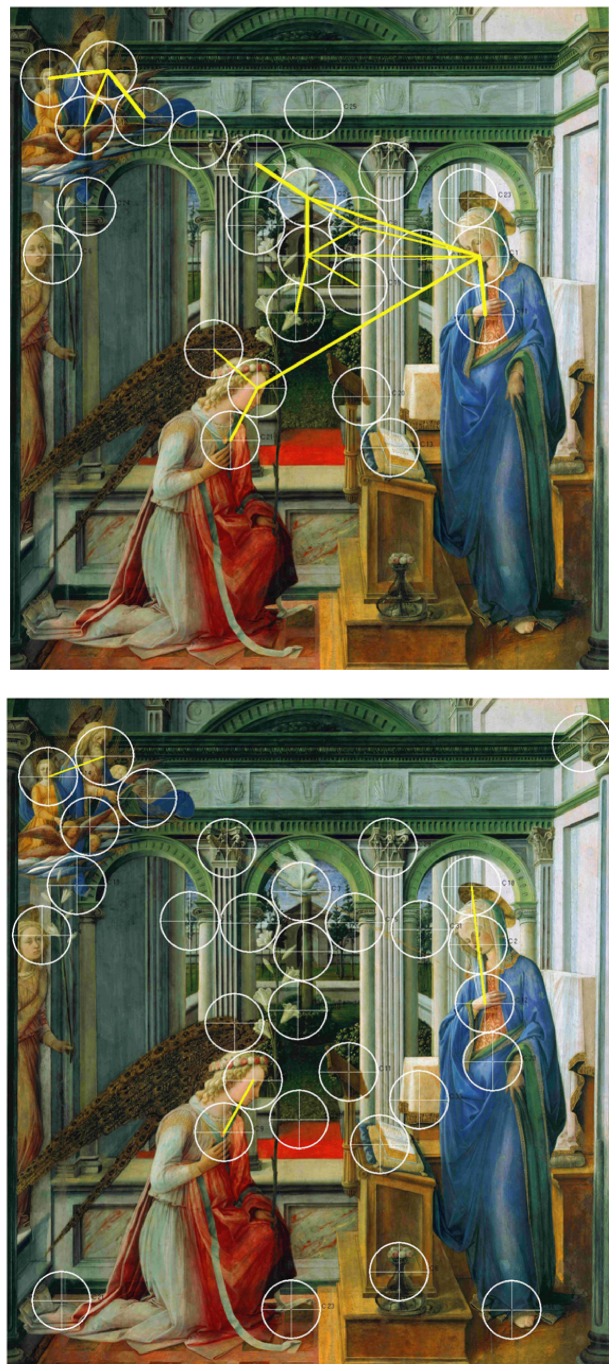
Speaking effects on frequently repeated transitions between fixation clusters – Lippi. Frequently repeated transitions between fixation clusters of the experimental group during minute 11 to 15. Cluster size: 60 mm. Frequently repeated transitions between fixation clusters of the control group during minute 11 to 15. Cluster size: 60 mm.

**Figure 7:**
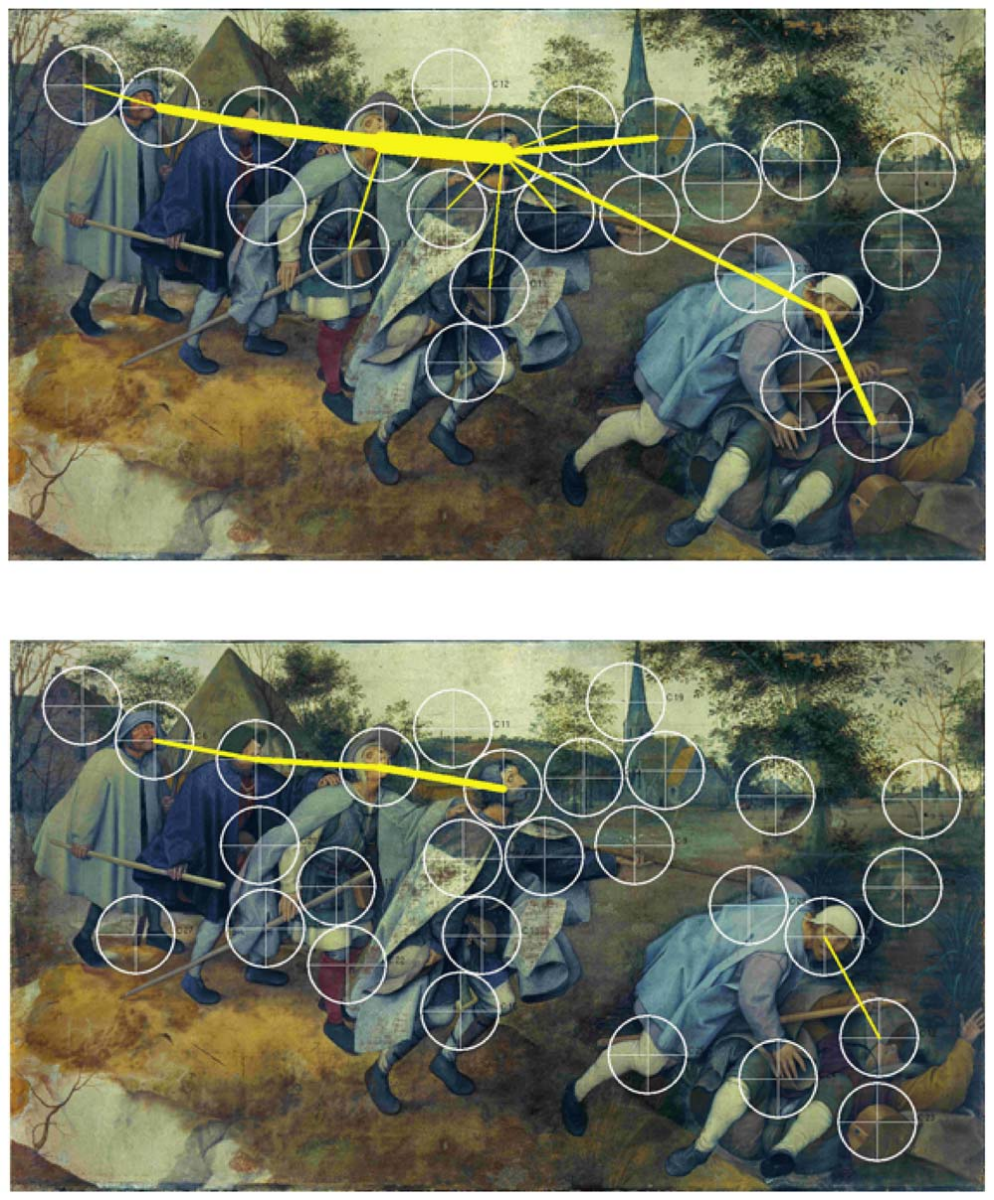
Speaking effects on frequently repeated transitions between fixation clusters – Bruegel. Upper part: Frequently repeated transitions between fixation clusters of the experimental group during minute 11 to 15. Cluster size: 60 mm. Lower part: Frequently repeated transitions between fixation clusters of the control group during minute 11 to 15. Cluster size: 60 mm.

**Figure 8:**
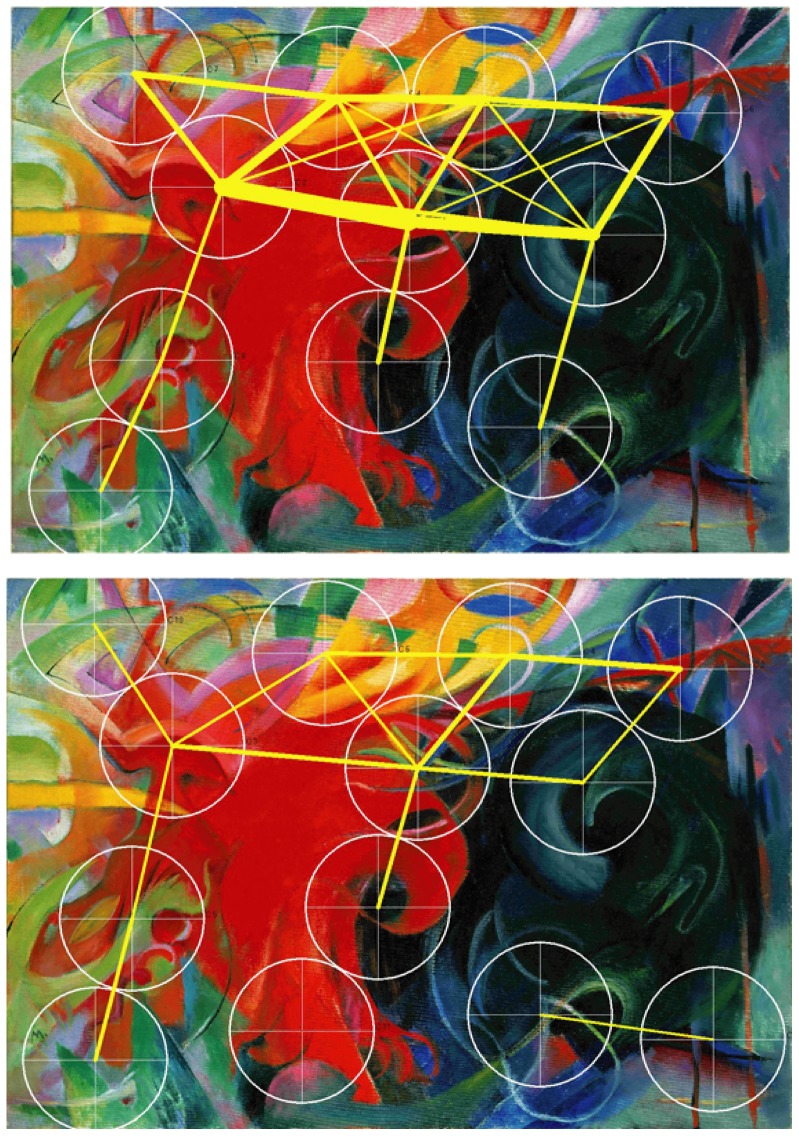
Speaking effects on frequently repeated transitions between fixation clusters – Marc. Upper part: Frequently repeated transitions between fixation clusters of the experimental group during minute 11 to 15. Cluster size: 120 mm. Lower part: Frequently repeated transitions between fixation clusters of the control group during minute 11 to 15. Cluster size: 120 mm.

**Figure 9:**
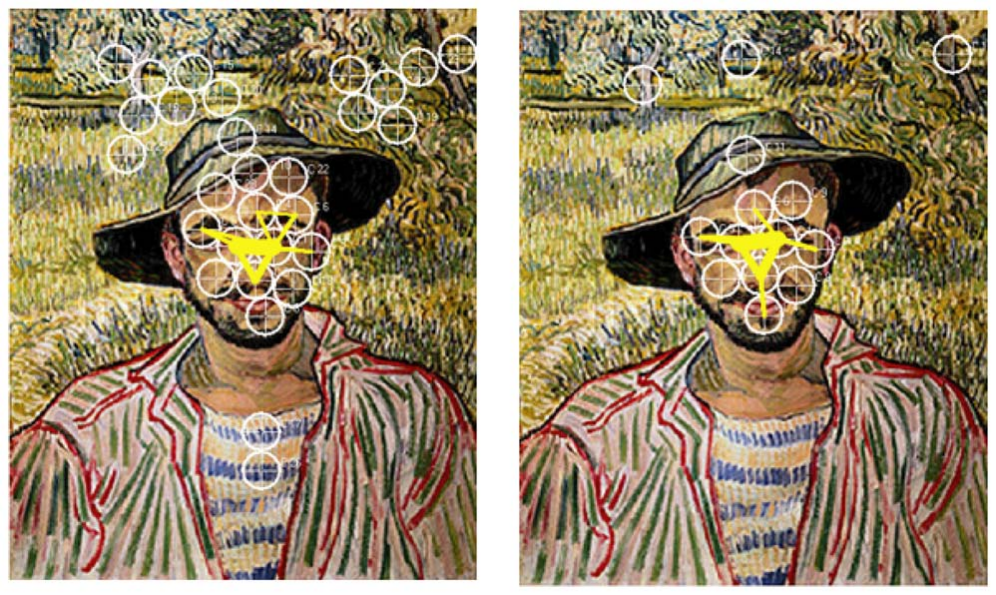
Speaking effects on frequently repeated transitions between fixation clusters – van Gogh. Left side: Frequently repeated transitions between fixation clusters of the experimental group during minute 11 to 15. Cluster size: 20 mm. Right side: Frequently repeated transitions between fixation clusters of the control group during minute 11 to 15. Cluster size: 20 mm.

## Results

While there was no significant difference between the experimental and control GROUPS during the first ten minutes of beholding, large speaking-related effects were found for all fixation- and gaze movement-related parameters during the final five minutes of beholding. This was reflected by the generally significant SEGMENT by GROUP interactions (henceforth labelled as “SxG”). Unless reported otherwise, speaking effects did not differ between paintings, as indicated by the almost exclusively non-significant interaction of PAINTING * SEGMENT * GROUP (“PxSxG”).

As can be seen in **Table 1**, the number of fixations frequencies increased (SxG: F_2,120_ = 14.3, p<.0001), and the mean duration of fixations decreased sharply during speaking (SxG: F_2,120_ = 35.3, p<.0001; see also **Figs. 2** **–** **5**), with no significant differences between the 5 min recording segments in controls. Furthermore, speaking reduced for all paintings the area covered by fixations (SxG: F_2,120_ = 6.0, p<.01). Gaze movement lengths, by contrast, increased with speaking (SxG: F_2,120_ = 58.3, p<.0001).

**Table 1:** Speaking effects on fixations, saccade lengths and cluster transitions.

		No Speaking			Speaking		
	IESG	Seg-1	Seg-2	Seg-3	Seg-1	Seg-2	Seg-3
**Fixations**							
number/min	14.3	154±17	150±15	148±15	157±13	150±14	162±13
mean duration	35.3	326±48	327±49	326±47	312±39	321±42	268±25
area covered	6.0	21±3	20±3	20±3	21±3	20±3	17±6
**Gaze**							
mean length	58.3	123±15	120±17	123±21	134±19	130±18	171±25
**Cluster transitions**							
number/min	29.8	52±8	50±7	51±8	52±9	49±9	63±11
mean length	59.6	98±12	95±15	99±19	110±19	103±17	145±27
percentage	10.2	34±4	34±4	35±4	34±4	33±5	39±7

*Note*: IESG = F-value of interaction effect SEGMENT x GROUP, all ps<.0001. Fixations: “number/min”: number of fixations per minute in a given data segment; “mean duration”: the average duration of fixations in a given data segment; “area covered”: the proportion of a painting's area that is covered by fixations (in percent); Gaze: “mean length”: gaze movements' average length (in mm of the panting per segment); Cluster transitions: “number/min”: absolute frequency of repeated cluster transitions in segment per minute; “mean length”: average length of repeated cluster transitions in segment (in mm of the painting); “percentage”: relative frequency of repeated cluster transitions in segment. Group averages across all four paintings are reported.

Constricting the entire set of gaze movements to those that connect fixation clusters, revealed strong increases in the number of repeated cluster transitions by speaking (SxG: F_2,120_ = 29.8, p<.0001). This effect was present for Lippi's (see **Fig. 6**), Bruegel's (see **Fig. 7**) and Marc's paintings (see **Fig. 8**), but not for van Gogh's (see **Fig. 9**) where from the beginning on most fixations and saccades concentrated on the face, in particular, on eyes and nose. These effects were highly significant for all paintings (ps<.001) except van Gogh (p = .13; PxSxG: F_6,360_ = 4.5, p<.001) and were exclusively due to significant differences in the experimental group between the second and the third recording segment. Calculating the number of repeated cluster transitions as a percentage of the overall gaze movements, revealed, that speaking increased this aspect of the gaze movement behaviour as well (SxG: F_2,120_ = 10.2, p<.0001). However, this effect was significant (ps<.01) only for the two paintings with a salient structure (or a “structural skeleton” [30]), that is, clear links between two or more salient objects (in the case of Lippi's painting: links between Godfather, Mary, the arch angle Gabriel (see **Fig. 6**), in Bruegel's case: the chain of blinds (see **Fig. 8**)). The same effect was not found for van Gogh's and Marc's paintings (PxSxG: F_6,360_ = 2.8, p<.10). Speaking also increased the mean length of repeated cluster transitions (SxG: F_2,120_ = 60.0, p<.0001) for all four paintings.

## Discussion

As outlined in the introduction, the investigation of speaking effects on gaze movements whilst looking at paintings is of greatest importance from the art-historical point of view as speaking can be an integral part of the contemplation itself, and hypothetical discourses between beholders have become a literary genre in art history. Classes of art history will often spend more than an hour discussing about just one painting. Learning how to describe works of art is a central component of the art history courses and art historians tend to assume that “you only see what you describe”. For these reasons, the primary aim of the present inter-disciplinary experiment was the investigation of the effects of speaking on gaze movements during the contemplation of paintings under fairly “naturalistic” conditions.

We found that during speaking as compared to no-speaking, participants exhibited more, but shorter fixations that covered a smaller area of the painting; they also produced more and longer gaze movements – covering more spread out regions of the painting – and repeated more often transitions between (some of) the fixation clusters. These results can be considered as highly robust as they were found in a large, and with respect to art historical knowledge: heterogeneous, sample, using a typologically diverse set of paintings. Furthermore, most of the reported effects held for all of the presented paintings, irrespective of their genre, and may therefore be generalizable in an empirical sense and possibly rather basic in a theoretical sense.

Studying the contemplation of paintings under “naturalistic” conditions improves the ecological validity of the obtained results, but may compromise their internal validity. With regard to the present study, this is mainly due to the fact that speaking whilst contemplating a painting is a complex process. Speaking alone includes various components such as lexical access (in itself presumably a multiple stages process [31], [32]) and executive control (including planning, monitoring and the like [33], [34]), all of which require to some degree attention. This holds also for the voluntary interplay between fixation and saccades, if the information acquired through such gaze movements is in some way “task-relevant” [35]. That these processes – complex in themselves – have to be co-ordinated when participants are required to speak while at the same time look at the painting, might suggest a shortening of attentional resources due to dual (or multiple) task demands. However, finding reduced fixation durations and squeezed areas covered by fixations only seemingly supports this interpretation, as will be argued in the following.

Dual task effects are not inevitably associated with impaired task performance. On the contrary, dual task effects may be difficult to design experimentally (e.g., because the two tasks executed simultaneously tax different “resource pools”), may be reduced by fluctuating trade-offs between the two tasks, or disappear with practice [36]. And even dual-task facilitation (that is, improved performance under dual-task rather than single-task conditions) is not uncommon in experimental research (e.g. [37], [38]).

The overall pattern of changes induced by speaking in the present study suggests – if anything – dual task *facilitation* of speaking on looking at paintings. Gaze duration has been found to be inversely related to the codeability and frequency of spoken words [20], suggesting that lexical access protracts rather than shortens fixation durations. Conversely, shorter fixation durations can be found for less complex stimuli [39] or “smarter” participants [40], pointing to the “ease” of access to the visual information as the common denominator for the swiftness of fixations. That shorter fixations during speaking were accompanied by longer gazes, a reduction in the area covered by fixations and, most importantly, more frequent gaze transitions between fixation clusters, points to an overall pattern of findings in which the gaze becomes swifter, more selective and better structured through speaking, rather than attentionally depleted. Furthermore, it is the repetition of gaze transitions between fixation clusters that have been suggested from an art-historical point of view to reflect an understanding of a painting's structure [41], [42]. In line with this reasoning, we assume that while speaking the beholder concentrates on the structure of the painting more than s/he did beforehand.

But why may this be the case? – As a potential explanation, we suggest that during speaking about a currently contemplated painting, actualisation (retrieval) of knowledge from short-term and/or long-term memory for speaking is coupled with visual attention and gaze control. While we can assume that during the first 10 minutes of contemplation a mnemonic representation or “knowledge” of a given painting has been formed both under the control and the experimental condition, only during speaking was this representation retrieved and activated in working memory to guide speaking, which, in turns, guides the gaze movements. The fact that during speaking the average fixation duration became on average 50 ms shorter suggests that the gaze movements as a temporal-spatial series of re-fixations was guided and thus followed speech production as a series of concepts, relations etc. that unfolds linearly in time. According to this interpretation, the painting's components and their discovered relationships are hence looked at to “match” or “confirm” the content of the spoken words. In this sense, speaking about a painting on the basis its mnemonic representation structures how it is gazed at.

This interpretation of our effects of voluntary language *production* (through speaking) on looking at paintings following their extensive silent contemplation is, by and large, compatible with experimental work on the interaction of mental representations of scenes and language *perception* in the control of gaze movements [43]–[45].

## Limitations of the Present Study and Directions for Future Research

The present study is, to the best of our knowledge, the first to suggest that speaking about a painting whilst looking at it speeds up fixations and makes the gaze movements more focused and better structured. While this effect as such is robust statistically and with respect to the number of participants, their range of art expertise and the typological diversity of the shown paintings, its explanation clearly requires further and probably more “experimental” studies. A direct follow up of the present study could compare the effects of speaking about visible unfamiliar paintings under conditions preceded or not preceded by what we have called the “baseline period”, in order to test our hypothesis that it is the build-up of a mnemonic representation of the painting during the baseline period that guides speaking and, in turn, gazing. Also, the speaking instructions could be varied such that while some participants talk about the visible painting, others talk about topics unrelated to art and the seen painting. This comparison could potentially show that speaking about the painting produces dual-task facilitation whereas speaking about an unrelated topic produces dual-task interference (as shown, for instance, by longer fixations and less transitions between fixation clusters). Furthermore, detailed analyses of the relative timings of speech production and gaze movements should be undertaken to investigate who takes the “lead” under which conditions, the gaze or the language. It is plausible that in this regard the presence of a mnemonic representation of the painting (be it through a baseline period or familiarity with the painting) will favour speaking, whereas the lack of such representations will give the lead to exploratory gazing. Finally, gaze movement patterns during speaking vs non-speaking should be related to “external” criteria such as recall of paintings or (some of) their pictorial elements from long-term memory to determine their supposed relationship with the mnemonic representation of the painting [46].
